# Tropical Diseases

**Published:** 1899-09

**Authors:** 


**Tropical Diseases.** By Patrick Manson, M.D. Pp. xvi., 607. London: Cassell and Company, Limited. 1898.

This manual covers the whole field of the diseases of warm climates, and is of an eminently practical nature. Chapters are devoted to fevers, diseases of undetermined nature, abdominal diseases, infective granulomatous diseases, animal parasites, skin diseases, and local diseases of uncertain nature, such as goundou and ainhum. The subject of malaria is dealt with most fully and successfully. With regard to the part played by the mosquito in the etiology of malarial infection Dr. Manson, whilst not accepting the theory as proved, recognises its importance, and thinks that every effort should be made to establish or confute it. Hæmoglobinuric fever, concerning the etiology of which there has been much discussion, will, Dr. Manson thinks, be found to be dependent on a form of malarial parasite peculiar to itself. The book is profusely illustrated, and the coloured plates of the malaria parasites are very finely executed. We can recommend the work to all students and teachers of tropical medicine.

